# Recurrent speciation of a tomato yellow leaf curl geminivirus in Portugal by recombination

**DOI:** 10.1038/s41598-018-37971-z

**Published:** 2019-02-04

**Authors:** Elvira Fiallo-Olivé, Helena P. Trenado, Diamantina Louro, Jesús Navas-Castillo

**Affiliations:** 1 Instituto de Hortofruticultura Subtropical y Mediterránea “La Mayora”, Consejo Superior de Investigaciones Científicas - Universidad de Málaga (IHSM-CSIC-UMA), Avenida Dr. Wienberg s/n, 29750 Algarrobo-Costa, Málaga Spain; 2 Instituto Nacional dos Recursos Biológicos (INRB), Quinta do Marquês, Oeiras, Portugal

## Abstract

Recurrent evolution can involve interspecific interactions, recognized to play a primary role in the diversification and organization of life. Both in the plant and animal kingdoms, the recurrent formation of allopolyploid species has been described. In the virosphere, recombination between isolates of different species has been shown to be a source of speciation. In this work, complete genome analysis showed that speciation through recombination of an emergent DNA plant virus, tomato yellow leaf curl Malaga virus (genus *Begomovirus*, family *Geminiviridae*), has occurred independently in Portugal and Spain, confirming previous observations with tomato yellow leaf curl Axarquia virus, also originated independently in Spain and Italy. These results will guide future research to discover new cases of recurrent emergence of recombinant virus species in geographical areas where the putative parents co-exist or can be introduced. This will reveal the role that recurrent speciation through recombination plays in the evolution of the virosphere and will help to understand the consequences of this phenomenon on the diversification of life.

## Introduction

Recurrent evolution has been extensively studied at genomic, molecular and phenotypic levels^1^. In some cases, recurrent evolution involves interspecific interactions, recognized to have played a primary role in the diversification and organization of life. Both in the plant and animal kingdoms, numerous cases of recurrent formation of allopolyploid species have been reported^2,3^. On the other extreme of biological complexity, the virosphere, recombination between isolates of different species as source of speciation is also well documented. Recombination, a phenomenon frequently observed in certain DNA viruses, has been shown to play an essential role in geminivirus (family *Geminiviridae*) diversification and evolution^4,5^ and its contribution to emergence of new species is well documented^6–11^.

**Figure 1:**
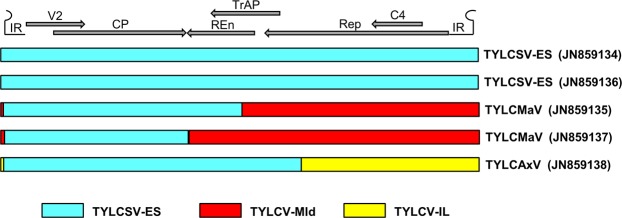
Genomic organization of the begomovirus isolates described in this work, belonging to the species *Tomato yellow leaf curl Sardinia virus* (TYLCSV), *Tomato yellow leaf curl Malaga virus* (TYLCMaV) and *Tomato yellow leaf curl Axarquia virus* (TYLCAxV). The origin of the recombinant fragments present in TYLCMaV and TYLCAxV isolates is indicated with different colors (blue, TYLCSV-ES; red, *Tomato yellow leaf curl virus*-Mld [TYLCV-Mld]; yellow, *Tomato yellow leaf curl virus*-IL [TYLCV-IL]). Arrows represent genes with the proteins products indicated: V2, movement-like protein; CP, coat protein; REn, replication enhancer protein; TrAP, transcription activator protein; Rep, replication-associated protein, C4, C4 protein. IR, intergenic region containing the origin of replication within a stem-loop structure.

Begomoviruses (genus *Begomovirus*, family *Geminiviridae*) are single-stranded DNA plant viruses with twin (geminate) virions consisting of two incomplete icosahedra transmitted by whiteflies (Hemiptera: Aleyrodidae) belonging to the *Bemisia tabaci* complex^12,13^. The genus *Begomovirus*, with more than 380 accepted species^12,14^, is the largest in the family and in the entire virosphere. Most begomoviruses have bipartite genomes consisting of two circular DNA components 2.5–2.8 kb in size, namely DNA-A and DNA-B. Monopartite begomoviruses have a single genomic component that resembles DNA-A of bipartite begomoviruses. The monopartite genomes encode the coat protein (CP) and a movement-like protein (V2) on the virus sense strand and a replication-associated protein (Rep), a transcription activator protein (TrAP), a replication enhancer protein (REn) and the C4 protein on the complementary sense strand (Fig. 1). Virus- and complementary-sense genes are separated by an intergenic region that contains the origin of replication within a stem-loop structure^12^.

Tomato yellow leaf curl disease (TYLCD) is caused by a complex of phylogenetically related begomoviruses that cause similar symptoms when infecting tomato plants. Infected plants are stunted, with leaflets rolled upwards and inwards and young leaves are slightly chlorotic, usually fruits are not produced or, if produced, are small and unmarketable. To date, thirteen begomovirus species have been officially recognized as being associated with TYLCD^12,14^, among them *Tomato yellow leaf curl virus* (TYLCV) that ranked third in the “Top 10 plant virus list” based on scientific and economic importance^15^. Most TYLCD-associated viruses (TYLCVs) have a monopartite genome. Mixed infections of different TYLCVs are frequent in epidemics worldwide^7,16–20^. The epidemics of TYLCD in the western Mediterranean basin constitute a paradigmatic example of this situation^21^. In Spain, the first reports of infections by TYLCVs were from the early 1990s, associated with the presence of the Spain (ES) strain of *Tomato yellow leaf curl Sardinia virus* (TYLCSV)^22^. Later, isolates of the Mild (Mld) and type [aka, and henceforth, Israel (IL)] strains of TYLCV were introduced in the country^23–25^. A few years after the detection of TYLCV, two novel recombinant viruses emerged: tomato yellow leaf curl Malaga virus (TYLCMaV) (as a result of a genetic exchange between isolates of the ES strain of TYLCSV and of the Mld strain of TYLCV)^9,26^ and tomato yellow leaf curl Axarquia virus (TYLCAxV) (as a result of a genetic exchange between isolates of the ES strain of TYLCSV and the IL strain of TYLCV)^7^. Both recombinant begomoviruses exhibited biological properties that suggested an ecological fitness higher than either parental begomovirus, including a wider host range, thus supporting the consideration of these recombinant viruses as *bona fide* species^14^. Recombinant TYLCVs have also been detected associated with mixed infections of the parental viruses in Italy, Jordan and Morocco^16,27–29^. TYLCMaV from Morocco and TYLCAxV from Jordan have been probably introduced from Spain and Italy, respectively. However, the TYLCAxV isolates from Italy have been originated by recombination between Italian TYLCV and TYLCSV isolates^16^. Also, sequencing of the intergenic region showed the presence of recombinant begomoviruses, involving TYLCV and TYLCSV as parents, in tomato and *S. nigrum* plants in Tunisia^30^, although a correct taxonomic adscription of them awaits the availability of complete viral sequences.

**Figure 2:**
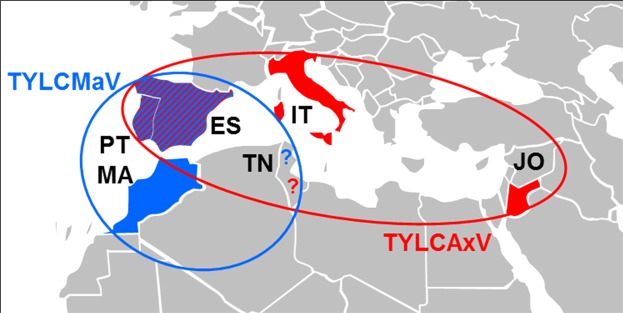
Map of the Mediterranean basin showing the countries where the recombinant species of the tomato yellow leaf curl virus complex, *Tomato yellow leaf curl Malaga virus* (TYLCMaV) (in blue) and *Tomato yellow leaf curl Axarquia virus* (TYLCAxV) (in red) have been reported to date. ES, Spain; IT, Italy; JO, Jordania; MA, Morocco; PT, Portugal; TN, Tunisia.?, to be confirmed by complete genome sequencing.

The TYLCMaV and TYLCAxV isolates characterized in the Mediterranean basin and close regions share a number of features: i) contain two recombinant fragments of similar length originated from each parental begomovirus (TYLCV and TYLCSV), ii) one of the recombination break points is close to the conserved stem-loop contained in the intergenic region and iii) the presence of a moiety containing the virion-sense genes originated from TYLCSV and a moiety containing the complementary-sense genes originated from TYLCV. Geographical distribution of TYLCV-TYLCSV recombinant begomoviruses is shown in Fig. 2.

In Portugal, although both strains of TYLCV, IL and Mld, seem to be present^31,32^, only one complete genome sequence of TYLCV-Mld was available when this work was initiated. The presence of TYLCSV in Portugal, however, has not been described to date. Unpublished data available in our laboratory suggested the presence of mixed infections of TYLCV and TYLCSV and/or recombinants originating from them in southern Portugal (Faro district, formerly Algarve province). This was based on molecular hybridization with specific probes of DNA extracts of some symptomatic tomato plants. In this work, we revisited our preliminary results and extended the analysis to a high number of tomato samples as well as to symptomatic samples of *S. nigrum* and other solanaceous weed species frequently associated with tomato crops, confirming the presence of the recombinant begomoviruses TYLCMaV and TYLCAxV in Portugal. Genealogical analysis suggested that TYLCAxV isolates from Portugal have been introduced from Spain. In contrast, the Portuguese TYLCMaV isolates have most probably originated independently from the Spanish isolates, thus providing strong evidence for the recurrent generation and establishment of this begomovirus species in different areas and periods of time.

## Results and Discussion

### TYLCV and TYLCSV are widely spread in southern Portugal

Two hundred sixty-three out of 350 tomato (75.1%) and 10 out of 28 weed (35.7%) samples were shown to be infected by begomoviruses belonging to the tomato yellow leaf curl virus complex after dot-blot molecular hybridization with probes specific for TYLCSV and TYLCV, respectively (Table 1, Supplementary Tables S1 and S2). Two hundred thirty-three tomato samples were positive for only one probe (16 with TYLCSV and 217 with TYLCV). Thirty tomato samples were positive with both probes. Nine out of 19 *S. nigrum* samples were positive for the TYLCV probe, two of them were also positive for the TYLCSV probe. The only *D. stramonium* sample analyzed was positive for the TYLCV probe. The existence of samples that hybridized both probes is consistent with mixed infections and/or with the presence of recombinant begomoviruses containing a recombination breakpoint within the intergenic region contained in the probes^7,9,33^.

**Table 1:** Dot-blot hybridization results using digoxigenin-labeled probes specific to the IR of TYLCSV and TYLCV.

Hybridization results		Number of samples				
TYLCSV probe	TYLCV probe	Tomato	*Solanum nigrum*	*Datura stramonium*	*Physalis ixocarpa*	*Salpichroa origanifolia*
−	−	87	10	0	6	2
+	−	16	0	0	0	0
−	+	217	7	1	0	0
+	+	30	2	0	0	0

See Supplementary Tables S1 and S2 for details on the samples.

PCR using primers specific for TYLCMaV and TYLCAxV was carried out with the 32 samples positive for both probes in the hybridization analysis. PCR using TYLCMaV specific primers (i.e. forward primer specific for TYLCV-Mld and reverse primer for TYLCSV-ES) showed amplicons from two tomato samples [150301/19–2 (#2) and 150301/19-6 (#6)]. The TYLCAxV specific primers (i.e. forward primer specific for TYLCV-IL and reverse primer for TYLCSV-ES) showed amplification in one *S. nigrum* sample [300902/38 (#38)]. Digestion of the rolling-circle amplification (RCA) product with *Bam*HI yielded two fragments of approximately 2.8 kbp with barely distinguishable electrophoretic mobilities in each tomato sample and one fragment in the *S. nigrum* sample, also of approximately 2.8 kbp. These fragments, each putatively corresponding to a full-length monopartite begomovirus genome, were cloned and sequenced. Nucleotide sequence analysis of two clones from tomato samples #2 (2776 nt, Genbank accession number JN859134) and #6 (2776 nt, JN859136) confirmed the presence of a monopartite begomovirus in both samples, showing the highest nucleotide identities (98.8% and 99.2%, respectively) with a TYLCSV-ES isolate from Spain (AJ519675). The two clones showed a nucleotide identity of 99.5% between them. Other clones from samples #2 (2782 nt, JN859135) and #6 (2782 nt, JN859137) showed the highest nucleotide identities (97.1% and 98.5%, respectively) with a TYLCMaV isolate from Spain (AF271234)^9^. Both clones showed a nucleotide identity of 97.3% between them. *S. nigrum* sample #38 was also infected by a begomovirus (2772 nt, JN859138) that showed the highest nucleotide identities with TYLCAxV isolates infecting *S. nigrum* in Spain (99.4%, AY227892)^7^ and tomato in Italy (92.4%, EU734831)^16^. In accordance with begomovirus species demarcation and nomenclature^14^, the sequences obtained in this work were isolates of TYLCSV, TYLCMaV and TYLCAxV for which the followings names were proposed: tomato yellow leaf curl Sardinia virus-Spain[Portugal-Algarve2-2001] (TYLCSV-ES[PT-Alg2-01]) and tomato yellow leaf curl Malaga virus-[Portugal-Algarve2-2001] (TYLCMaV-[PT-Alg2-01]) for isolates from sample #2, tomato yellow leaf curl Sardinia virus-Spain[Portugal-Algarve6-2001] (TYLCSV-ES[PT-Alg6-01]) and tomato yellow leaf curl Malaga virus-[Portugal-Algarve6-2001] (TYLCMaV-[PT-Alg6-01]) for isolates from sample #6, and tomato yellow leaf curl Axarquia virus-Spain [Portugal-Algarve38-Solanum nigrum-2002] (TYLCVAxV-ES[PT-Alg38-Sn-02]) for isolate from sample #38.

Recombination analysis of the TYLCMaV and TYLCAxV isolates from Portugal obtained in this work confirmed their recombinant nature, as suggested by the high nucleotide identity showed with isolates of those begomoviruses from Spain that are known to be recombinants^7,9^. Isolate TYLCMaV-[PT-Alg2-01] showed a recombination event involving TYLCV-Mld (AF105975) as major parent and TYLCSV-ES[PT-Alg2-01] as minor parent (Fig. 1, Table 2). Isolate TYLCMaV-[PT-Alg6-01] showed a recombination event involving TYLCV-Mld (AF105975) as major parent and TYLCSV-ES[PT-Alg6-01] as minor parent. However, TYLCAxV from Portugal showed a recombination event involving two isolates from Spain: TYLCSV-ES (Z25751) as major parent and TYLCV-IL (AJ489258) as minor parent (Fig. 1, Table 2). One of the recombination breakpoints of all three isolates was located in the stem-loop sequence conserved in the IR. It is known that this stem-loop is a hot-spot for recombination in begomoviruses and other geminiviruses^8,34^.

**Table 2:** Recombination analysis of TYLCMaV and TYLCAxV.

Recombinant virus	Recombination breakpoints	Parent sequences		Methods that detected recombination*	Lowest *p*-value
		Major	Minor		
TYLCMaV (JN859135)	5-1400	TYLCV-Mld (AF105975)	TYLCSV-ES (JN859134)	R, G, B, M, C, 3 S	2.927 × 10^−141^
TYLCMaV (JN859137)	17-1088	TYLCV-Mld (AF105975)	TYLCSV-ES (JN859136)	R, G, B, M, C, 3 S	5.524 × 10^−115^
TYLCAxV (JN859138)	1740-4	TYLCSV-ES (Z25751)	TYLCV-IL (AJ489258)	R, G, B, M, C, S, 3 S	1.400 × 10^−151^

RDP4 package^40^ was used for detecting recombination events in the TYLCMaV and TYLCAxV sequences obtained in this work. *R, RDP; G, GENCONV; B, BootScan (B); M, MaxChi; C, Chimaera; S, SiScan and 3 S, 3Seq. The method with the lowest *p*-value obtained for each recombination event is underlined.

To our knowledge, this is the first report confirming the presence of TYLCSV, TYLCMaV and TYLCAxV in Portugal, where TYLCV was the only begomovirus associated with TYLCD to date^31,32^.

### TYLCMaV, but not TYLCAxV, originated in Portugal

**Figure 3:**
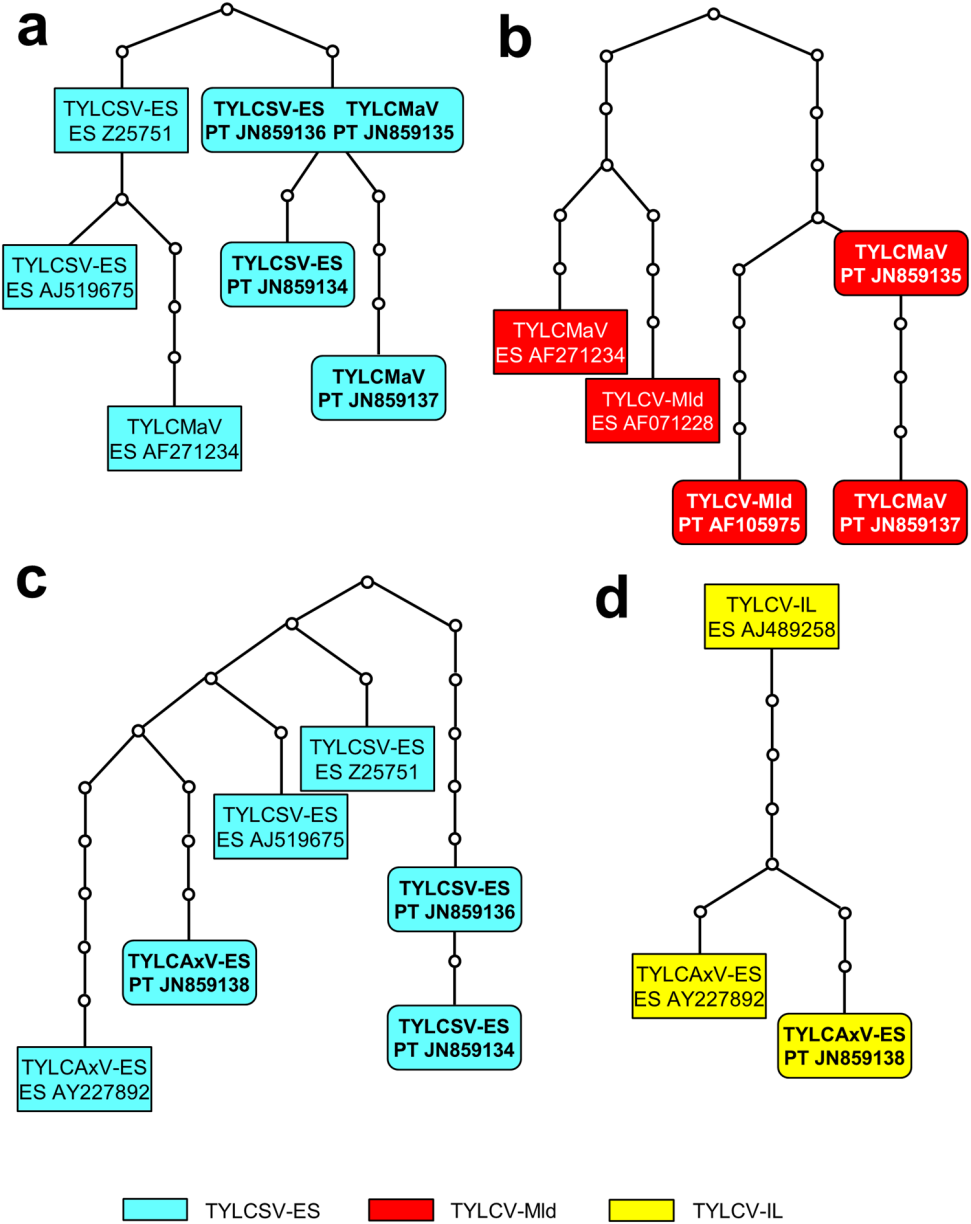
Genealogical relationships between the sequences of the TYLCSV (**a,c**) and TYLCV (**b,d**) moieties of the recombinant begomoviruses TYLCMaV (**a,b**) and TYLCAxV (**c,d**) found in Portugal and Spain, and their parental viruses (TYLCSV-ES, TYLCV-Mld and TYLCV-IL). Maximum parsimony networks at 95% connection limit were constructed with the TCS program^43^ and identify both the relationships between the begomovirus sequences and the number of nucleotide substitutions connecting them. The small circles indicate mutational changes between the linked sequences. The sequences from Portugal are in bold inside rounded rectangles.

**Figure 4:**
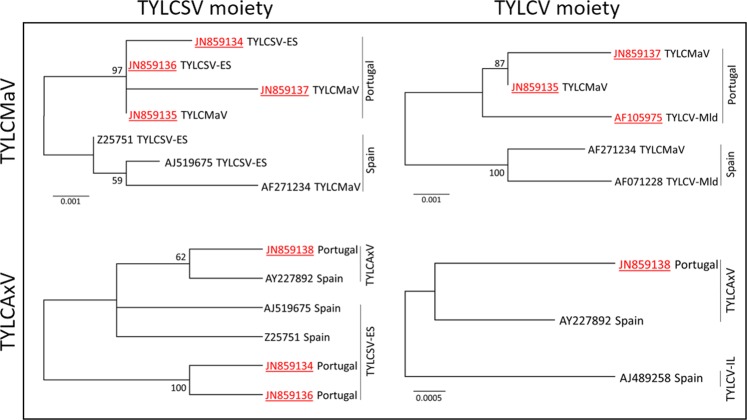
Phylogenetic relationships between the sequences of the TYLCSV (left trees) and TYLCV (right trees) moieties of the recombinant begomoviruses TYLCMaV (upper trees) and TYLCAxV (lower trees) found in Portugal and Spain, and their parental viruses (TYLCSV-ES, TYLCV-Mld and TYLCV-IL). The trees were constructed by the maximum-likelihood method (500 replicates) with the MEGA X program using the best-fit model, T92. The tree for the TYLCSV moiety of TYLCAxV was condensed with a cutoff value of 50%. The tree for the TYLCV moiety of TYLCAxV could not be submitted to bootstrapping because only three sequences are available. The bars below the trees indicate nucleotide substitutions per site. The sequences from Portugal are in red and underlined.

TYLCMaV is a species arisen from recombination between TYLCSV-ES and TYLCV-Mld (Table 2, Supplementary Fig. S1). Despite TYLCMaV sequences from Portugal having high nucleotide identity with the Spanish isolate, the moiety of the TYLCMaV genomes from Portugal originated from TYLCSV were more similar to TYLCSV-ES isolates from Portugal (0–4 nt substitutions) than to TYLCSV-ES/TYLCMaV isolates from Spain (4–13 nt substitutions), as was shown by the genealogical network analysis (Fig. 3a). Also, the moiety of the TYLCMaV genomes from Portugal originated from TYLCV-Mld were more similar to TYLCV-Mld isolates from Portugal (6–10 nt substitutions) than to TYLCV-Mld/TYLCMaV isolates from Spain (11–16 nt substitutions) (Fig. 3b). This strongly suggests that the TYLCMaV isolates characterized in this work were not introduced from Spain but arose in Portugal as a result of an independent recombination event. In contrast, comparison of the moiety of the Portuguese TYLCAxV isolate (arisen from recombination between TYLCSV-ES and TYLCV-IL; Table 2, Supplementary Fig. S1) that originated from TYLCSV-ES showed that was more similar to TYLCSV-ES/TYLCAxV isolates from Spain (7–10 nt substitutions) than to TYLCSV-ES isolates from Portugal (13–15 nt substitutions) (Fig. 3c), thus suggesting that TYLCAxV most probably emerged in Spain and moved to Portugal, where it was also found infecting *S. nigrum*, a wild plant widely distributed in the Mediterranean basin that has been suggested to be an optimal niche for the development of recombinant begomoviruses^7^. This hypothesis was reinforced by the fact that TYLCV-IL, one of the parents of TYLCAxV, has never been reported in the Faro district in southern Portugal. The only report of TYLCV-IL in this country was based on a partial sequence from Portalegre, a district in central Portugal^31^. The results of the genealogical network analysis were confirmed by phylogenetical analysis (Fig. 4). Thus, both moieties of the Portuguese TYLCMaV isolates were clustered with the putative parental begomovirus isolates from Portugal (TYLCSV-ES, TYLCV-Mld); in contrast, the Portuguese TYLCAxV isolate clustered with the TYLCAxV isolate from Spain.

Sequence comparisons and phylogenetic analysis have shown that Italian TYLCAxV isolates [classified within Sicily1 (Sic1) or Sicily2 (Sic2) strains; Supplementary Fig. S1, Table 3] originated from parental viruses present in Italy, including the Sicily (Sic) strain of TYLCSV^16,27^, thus providing additional evidence for recurrent speciation in the TYLCV complex. In contrast, the TYLCAxV isolates from Jordania^28^ and the TYLCMaV isolates from Morocco^29^ were very probably introduced from Italy and Spain, respectively (Supplementary Fig. S1).

**Table 3:** Begomoviruses of the TYLCV complex from the Mediterranean basin analyzed in this work.

Virus	Strain	Parental viruses	Country	GenBank acc. number
TYLCAxV	Spain (ES)	TYLCV-IL/TYLCSV-ES	Spain	AY227892
			Portugal	JN859138*
	Sicily1 (Sic1)	TYLCV-IL/TYLCSV-Sic	Italy	EU734831
	Sicily2 (Sic2)	TYLCV-IL/TYLCSV^#^	Italy	EU734832
			Jordan	KM215610
TYLCMaV		TYLCV-Mld/TYLCV-ES	Spain	AF271234
			Portugal	JN859135*
			Portugal	JN859137*
			Morocco	LN846611
			Morocco	LN846612
TYLCSV	Sardinia (Sar)		Italy	X61153
			Italy	GU951759
			Jordan	JX131285
	Sicily (Sic)		Italy	Z28390
	Spain (ES)		Spain	L27708
			Spain	Z25751
			Spain	AJ519675
			Portugal	JN859134*
			Portugal	JN859136*
TYLCV	Israel (IL)		Spain	AJ489258
			Italy	DQ144621
			Jordan	EF433426
	Mild (Mld)		Spain	AF071228
			Portugal	AF105975
			Spain	AJ519441

*Sequences obtained in this work. ^#^It could not be determined if one of the parentals was TYLCSV-Sic or TYLCSV-Sar^16^.

The emergence of TYLCMaV isolates in Portugal as the result of recombination between the parental begomoviruses TYLCV-Mld and TYLCSV, a phenomenon previously described in Spain^9^, suggests that wherever the parental viruses have contact through mixed infections, the same recombinant virus will probably be generated. Both TYLCMaV and TYLCAxV isolates generated in Spain exhibited higher ecological fitness than either parental virus, including a wider host range^7,9^. In this regard, Spanish TYLCMaV isolates, that were initially detected in tomato plants, were found later in common bean crops where they displaced the parental begomoviruses^9^. This behavior highlights the relevance of the recurrent generation of recombinant begomoviruses causing the TYLCD in new geographical areas and agrosystems.

The results obtained in this work will guide future research to discover new cases of recurrent appearance and emergence of recombinant virus species in geographical areas where the putative parents co-exist or can be introduced, which is highly probable not only in the case of geminiviruses but in other plant, animal and human viruses having highly recombinogenic genomes. This will reveal the actual role that recurrent speciation through recombination plays in the evolution of the virosphere and will help to understand the consequences of this phenomenon on the diversification of life as a whole.

## Materials and Methods

### Plant material, DNA extraction and molecular hybridization

Tomato (n = 350, Supplementary Table S1) and solanaceous weed (n = 28, Supplementary Table S2) plants were collected from different locations of Faro district, the southernmost district of Portugal and coincident with the historical Algarve province, from 2000 to 2005. All samples showed symptoms resembling those caused by TYLCD. Total DNA extracts were obtained from leaf samples^35^ and kept at −20 °C until they were used for dot-blot hybridization. For that, 1 μL of DNA extract from each sample was spotted on a positively charged nylon membrane (Roche Diagnostics, Mannheim, Germany). Squash-blotted cross-sectioned leaf petioles of healthy and TYLCMaV- or TYLCAxV-infected tomato (cv. Moneymaker) plants were used as negative and positive controls, respectively. Digoxigenin (DIG)-labeled DNA probes were prepared by polymerase chain reaction (PCR) containing sequences of the IR of TYLCSV and TYLCV, respectively^25^ (Table 4). The probes were prepared by PCR according to the DIG-labeling detection kit (Roche Diagnostics). Hybridization was carried out under high stringency conditions (washing steps at 65 °C in 0.1x SSC [15 mM NaCl and 1.5 mM sodium citrate] and 0.1% sodium dodecyl sulfate) following standard procedures. Hybridization signals were detected on X-ray film after treatment with CDP-Star (Roche Diagnostics).

**Table 4:** Primers used to generate DNA probes specific to the IR of TYLCSV and TYLCV.

Begomovirus [isolate]	GenBank acc. number	Primers (5′-3′)	Probe coordinates*	Reference
TYLCSV-ES [ES-Mur1-92]	Z25751	MA-14 (TGCATTTATTTGAAAACG) MA-15 (AAAGGATCCCACATATTG)	2580–155	^25^
TYLCV-Mld [ES-72-97]	AF071228	MA-30 (GAGCACTTAGGATATGTGAGG) MA-31 (AGTGGATCCCACATATTGC)	2557–161	^25^

*Nucleotide coordinates considering as first nucleotide that after the nicking site within the conserved nonanucleotide at the origin of replication.

### PCR, rolling-circle amplification (RCA) and cloning

Plant samples that were positive for both probes were analyzed by PCR using the primers MA117 (5′-TAAGGAGCACTTAGGATATG-3′) and MA116 (5′-GTAGGGCCCACTACTTTATC-3′) specific for TYLCMaV^9^ and MA250 (5′-GGTGTCCCTCAAAGCTCTATGGCAATCG-3′) and MA116 specific for TYLCAxV^7^. PCR reactions were carried out using BIOTAQ DNA polymerase (Bioline, London, UK). The amplification program started with an initial denaturing step at 94 °C for 2 min, followed by 30 cycles of 94 °C for 1 min, 50 °C for 1 min and 72 °C for 1 min, plus a last elongation step at 72 °C for 5 min. DNA from selected samples was used as template in RCA reactions using φ29 DNA polymerase (TempliPhi kit, GE Healthcare). RCA products were digested with *Bam*HI. Selected fragments of approximately 2.8 kb were cloned in pBluescript II SK(+) and transformed in *Escherichia coli* DH5α by electroporation. Clones of expected size were automatically sequenced (Macrogen Inc., Seoul, South Korea).

### Sequence analysis

The BLAST program (https://blast.ncbi.nlm.nih.gov/Blast.cgi) was used for initial sequence similarity searches. Selected sequences were aligned with MUSCLE^36^ and pairwise identity scores were calculated with SDT (Sequence Demarcation Tool)^37^. Phylogenetic evidence of recombination was revealed by using the neighbor-net method in the program SPLITSTREE4^38^ and statistically verified by using the pairwise homoplasy index (PHI) test^39^. The identification of potential recombinant fragments was carried out using the nine methods included in the RDP4 package with default settings (primary scan with RDP, GeneConv and MaxChi and secondary scan with BootScan and SiScan)^40^ from the alignment generated by CLUSTAL X (v.2.07)^41^. The common sequences of the TYLCSV and TYLCV moieties identified in the recombinant begomoviruses were analyzed using statistical parsimony^42^ with the program TCS (v.1.21)^43^. The resulting genealogical networks identify both the relationship between the different sequences as well as the number of nucleotide substitutions connecting them. Both begomovirus moieties were also submitted to phylogenetic analysis using maximum likelihood (ML) after selecting the best-fit model of nucleotide substitution based on the corrected Akaike information criterion (AICc) and Bayesian information criterion (BIC) as implemented in MEGA X^44^. Details of the begomoviruses belonging to the TYLCV complex analyzed, included those characterized in this work, are given in Table 3.

## Supplementary information

Supplementary material
