# Supplementary material for: Recurrent speciation of a tomato yellow leaf curl geminivirus in Portugal by recombination
Source: Sci Rep. 2019 Feb 4;9:1332. doi: 10.1038/s41598-018-37971-z (PMC6362282; doi:10.1038/s41598-018-37971-z)

Recurrent speciation of a tomato yellow leaf curl geminivirus in Portugal by recombination

Elvira Fiallo-Olivé[1], Helena P. Trenado[1], Diamantina Louro[2] & Jesús Navas-Castillo[1]*

[1]Instituto de Hortofruticultura Subtropical y Mediterránea "La Mayora", Consejo Superior de Investigaciones Científicas - Universidad de Málaga (IHSM-CSIC-UMA), Avenida Dr. Wienberg s/n, 29750 Algarrobo-Costa, Málaga, Spain.
[2]Instituto Nacional dos Recursos Biológicos (INRB), Quinta do Marquês, Oeiras, Portugal.

*Corresponding author (email: jnavas@eelm.csic.es)

- Supplementary Table S1
- Supplementary Table S2
- Supplementary Figure S1

Supplementary Table S1. Tomato samples collected in this work in southern Portugal and dot-blot hybridization results. Digoxigening-labeled probes specific to the IR of TYLCSV and TYLCV were used. *Sample codes are in the format DDMMYY/#. $CEHFP, Centro de Experimentação Horto-frutícola do Patacão, Faro.

Sample code*	Cultivar	Location	Probe	
			TYLCSV	TYLCV
281200/13	Daniela	CEHFP$	-	+
281200/16	Daniela	CEHFP	-	+
281200/18	Daniela	CEHFP	+	-
281200/25	Daniela	CEHFP	-	+
150301/15	Daniela	CEHFP	-	+
150301/19-1	Daniela	CEHFP	+	+
150301/19-2	Daniela	CEHFP	+	+
150301/19-4	Daniela	CEHFP	+	-
150301/19-5	Daniela	CEHFP	+	+
150301/19-6	Daniela	CEHFP	+	+
150301/25	Daniela	CEHFP	-	-
310701/1-5	unknown	São Bartolomeu de Messines	+	+
310701/1-7	Daniela	CEHFP	+	+
310801/1	unknown	Ludo	-	+
310801/2	unknown	Ludo	-	+
210901/18	Tavira	Patacão	-	+
210901/22	Bond	Patacão	-	+
210901/25	Bond	Patacão	-	+
210901/26	Manthus	Patacão	-	+
210901/27	Bond	Patacão	-	+
311001/1	Salmor	Pechão	-	+
311001/2	Salmor	Pechão	-	+
311001/4	Anastasia	Pechão	-	+
311001/5	Anastasia	Pechão	-	+
311001/8	Anastasia	Pechão	-	+
311001/9	Tavira	Pechão	+	+
311001/10	Tavira	Pechão	-	+
311001/11	Tavira	Pechão	-	+
311001/12	Tyrade	Estói	-	+
311001/13	Tyrade	Estói	-	+
311001/14	Tyrade	Estói	-	+
311001/18	Zynac	Estói	-	+
311001/19	Zynac	Estói	-	+
311001/20	Zynac	Estói	-	+
311001/22	Boro	Estói	-	+
311001/23	Boro	Estói	+	+
311001/24	Boro	Estói	-	+
311001/26	Boro	Estói	-	+
311001/28	unknown	Estrada da Penha	-	+
311001/29	unknown	Estrada da Penha	-	+
311001/30	unknown	Estrada da Penha	-	+
311001/31	unknown	Estrada da Penha	-	+
311001/32	unknown	Estrada da Penha	+	+
311001/34	Salmor	Estói	+	+
311001/35	Salmor	Estói	-	+
311001/37	Salmor	Estói	-	+
311001/51	Anastasia	Marim	-	+
311001/55	Anastasia	Marim	-	+
311001/66	Emily	Montenegro	-	+

311001/69	Salmor	Campina de Faro	+	+
311001/70	Salmor	Campina de Faro	-	+
311001/71	Boro	Campina de Faro	-	+
311001/72	Boro	Campina de Faro	-	+
311001/73	Boro	Campina de Faro	-	+
311001/76	Tavira	Estrada da Penha	-	+
311001/77	Tavira	Estrada da Penha	-	+
311001/78	Tavira	Estrada da Penha	-	+
311001/83	unknown	Estrada da Penha	-	-
070102/15	Salmor	Estói	-	+
070102/16	Salmor	Estói	-	+
070102/18	Salmor	Estói	-	+
070102/21	Salmor	Estói	-	+
070102/22	unknown	Estói	-	+
070102/23	unknown	Estói	-	+
070102/24	unknown	Estói	-	-
070102/25	unknown	Estói	-	-
070102/26	unknown	Estói	+	-
070102/37	unknown	Estrada da Penha	-	+
070102/38	unknown	Estrada da Penha	-	+
070102/40	unknown	Estrada da Penha	+	-
070102/41	unknown	Estrada da Penha	-	+
070102/42	unknown	Estrada da Penha	-	+
070102/43	unknown	Estrada da Penha	-	+
070102/44	unknown	Estrada da Penha	-	+
110202/2	Genaro	Estói	-	+
110202/3	Genaro	Estói	-	+
110202/4	Genaro	Estói	-	+
110202/18	Tavira	Estrada da Penha	-	+
110202/21	Tyrade	Estrada da Penha	-	+
110202/22	Tyrade	Estrada da Penha	-	+
110202/23	Tyrade	Estrada da Penha	-	-
110202/24	Tyrade	Estrada da Penha	-	+
110202/27	Tyrade	Estrada da Penha	-	+
110202/28	Tavira	Estrada da Penha	-	+
110202/29	Tavira	Estrada da Penha	-	+
110202/30	Tavira	Estrada da Penha	-	+
110202/32	Tavira	Estrada da Penha	-	+
110202/39	unknown	Estrada da Penha	-	+
110202/40	unknown	Estrada da Penha	-	+
110202/41	unknown	Estrada da Penha	-	+
110202/42	unknown	Estrada da Penha	-	+
250202/1	unknown	Luz de Tavira	-	+
250202/2	unknown	Luz de Tavira	-	+
250202/3	unknown	Luz de Tavira	-	+
250202/4	unknown	Luz de Tavira	-	+
250202/7	unknown	Luz de Tavira	-	+
250202/8	unknown	Luz de Tavira	-	+
140302/16	Genaro	Maragota	-	-
140302/57	Xénon	Olhão	-	+
140302/65-A	unknown	Olhão	+	+
150702/28	unknown	Chelote	-	+
150702/29	unknown	Chelote	+	+
150702/30	unknown	Chelote	-	+
150702/31	unknown	Chelote	-	+
150702/32	unknown	Chelote	-	+
150702/33	unknown	Chelote	-	+
150702/34	unknown	Chelote	-	+

150702/35	unknown	Chelote	-	+
150702/36	unknown	Chelote	-	+
150702/37	unknown	Chelote	-	+
300902/5	Tavira	Estrada da Penha	+	-
300902/8	Fa 179	Ferradeira	-	-
300902/10	Prevesa	Ferradeira	-	+
300902/13	Fa 179	Bela Salema	+	-
300902/14	Fa 179	Bela Salema	+	-
300902/15	Fa 179	Bela Salema	+	+
300902/18	Tavira	Estrada da Penha	-	+
300902/19-1	Cencara	Faro	-	+
300902/19-2	Cencara	Faro	+	+
300902/19-3	Cencara	Faro	-	+
300902/19-4	Cencara	Faro	-	+
300902/19-5	Cencara	Faro	-	+
300902/19-6	Cencara	Faro	-	+
300902/19-7	Cencara	Faro	+	-
300902/21-1	Tyrade	Faro	-	+
300902/21-2	Tyrade	Faro	-	+
300902/21-3	Tyrade	Faro	-	-
111102/1	Preveza	Montenegro	-	+
111102/2	Preveza	Montenegro	-	+
111102/6	Preveza	Montenegro	-	+
111102/7	Preveza	Montenegro	-	+
111102/9	Preveza	Montenegro	-	+
111102/14	Cencara	Patacão	-	+
111102/15	Cencara	Patacão	-	+
111102/17	Cencara	Patacão	-	+
111102/20	Sinatra	Patacão	+	+
111102/21	Sinatra	Patacão	-	+
111102/22	Sinatra	Patacão	-	+
111102/31	Cencara	Chelote	-	+
111102/32	Cencara	Chelote	+	+
111102/33	Cencara	Chelote	-	+
111102/38	Boro	CEHFP	-	+
111102/40	Boro	CEHFP	-	+
111102/42	Boro	CEHFP	-	+
111102/43	Boludo	CEHFP	-	+
111102/44	Boro	CEHFP	-	+
111102/45	Zynac	CEHFP	-	+
111102/46	Zynac	CEHFP	-	+
111102/47	Zynac	CEHFP	-	+
050303/1	Preveza	Montenegro	-	+
050303/4	Preveza	Montenegro	-	+
050303/8	Zynac	Montenegro	-	+
050303/12	unknown	Estrada da Penha	+	-
050303/13	unknown	Estrada da Penha	+	-
050303/14	unknown	Estrada da Penha	+	-
050303/15	unknown	Estrada da Penha	+	-
050303/21	Anastasia	CEHFP	-	-
050303/23	Birloque	CEHFP	-	-
050303/25	Boro	CEHFP	-	+
050303/26	Boro	CEHFP	-	+
050303/27	Boro	CEHFP	-	-
050303/28	Zynac	CEHFP	-	+
050303/29	Sinatra	CEHFP	-	+
050303/30	Sinatra	CEHFP	-	+
050303/32	Sinatra	CEHFP	-	+

050303/33	Sinatra	CEHFP	-	+
110504/107-17	unknown	Estrada da Penha	-	+
110504/107-19	unknown	Estrada da Penha	-	+
110504/107-21	unknown	Estrada da Penha	-	+
110504/107-23	unknown	Estrada da Penha	+	+
110504/110-1	Zinac	Porto Carro	-	-
110504/110-2	Zinac	Porto Carro	+	+
110504/110-3	Zinac	Porto Carro	+	+
110504/112	Tavira	Luz de Tavira	-	+
110504/113-5	Sinatra	Luz de Tavira	-	+
110504/113-7	Sinatra	Luz de Tavira	-	+
110504/113-8	Sinatra	Luz de Tavira	-	+
110504/113-10	Sinatra	Luz de Tavira	-	-
110504/113-11	Sinatra	Luz de Tavira	-	-
110504/114-A	Sinatra	Barrocal-Messines	-	+
110504/114-B	Sinatra	Barrocal-Messines	-	+
110504/114-14	Sinatra	Barrocal-Messines	-	+
110504/114-15	Sinatra	Barrocal-Messines	-	+
110504/114-16	Sinatra	Barrocal -Messines	-	+
071004/8	Birloque	CEHFP	-	+
071004/11	Birloque	CEHFP	-	+
071004/12	Birloque	CEHFP	-	+
071004/13	Anastasia	CEHFP	-	+
071004/14	Anastasia	CEHFP	-	+
071004/15	Anastasia	CEHFP	-	-
071004/16	Anastasia	CEHFP	-	+
071004/17	Anastasia	CEHFP	-	+
071004/18	Anastasia	CEHFP	-	+
071004/20	Anastasia	CEHFP	-	+
071004/21	Anastasia	CEHFP	-	+
071004/23	Zinac	CEHFP	-	+
071004/24	Zinac	CEHFP	+	+
071004/25	Zinac	CEHFP	-	+
071004/26	Zinac	CEHFP	-	+
071004/27	Zinac	CEHFP	-	+
071004/28	Zinac	CEHFP	-	+
071004/29	Zinac	CEHFP	-	+
071004/30	Zinac	CEHFP	-	+
071004/31	Zinac	CEHFP	-	-
071004/32	Zinac	CEHFP	-	-
081004/1	Sencara	Luz de Tavira	+	+
081004/2	Sencara	Luz de Tavira	-	+
081004/3	Sencara	Luz de Tavira	-	-
081004/4	Sencara	Luz de Tavira	-	+
081004/5	Sencara	Luz de Tavira	-	+
081004/6	Sencara	Luz de Tavira	-	-
081004/7	Sencara	Luz de Tavira	-	+
081004/8	Sencara	Luz de Tavira	-	+
081004/9	Sencara	Luz de Tavira	-	+
081004/10	Sencara	Luz de Tavira	-	-
081004/16	Patrona	Luz de Tavira	-	-
081004/17	Patrona	Luz de Tavira	-	-
081004/18	Patrona	Luz de Tavira	-	-
081004/19	Patrona	Luz de Tavira	-	+
081004/20	Patrona	Luz de Tavira	-	-
251004/1	Tyrade	CEHFP	-	+
251004/2	Tyrade	CEHFP	-	+
251004/3	Tyrade	CEHFP	-	-

251004/4	Tyrade	CEHFP	-	-
251004/5	Tyrade	CEHFP	-	-
251004/6	Birloque	CEHFP	-	-
251004/7	Birloque	CEHFP	-	-
251004/9	Birloque	CEHFP	-	-
251004/10	Birloque	CEHFP	-	-
251004/11	Birloque	CEHFP	-	-
251004/12	Birloque	CEHFP	-	-
251004/15	Anastasia	CEHFP	-	-
251004/16	Anastasia	CEHFP	-	-
251004/17	Anastasia	CEHFP	-	-
251004/18	Anastasia	CEHFP	-	-
251004/19	Anastasia	CEHFP	-	-
251004/21	Encanto	CEHFP	-	-
251004/22	Bybal	CEHFP	-	-
251004/23	Bybal	CEHFP	-	+
251004/27	Bybal	CEHFP	-	+
251004/28	Tybet	CEHFP	-	-
251004/29	Tybet	CEHFP	-	+
251004/30	Tybet	CEHFP	-	-
251004/31	Tybet	CEHFP	-	-
251004/32	Tybet	CEHFP	-	+
251004/33	Tybet	CEHFP	-	+
251004/34	Atyla	CEHFP	-	+
251004/35	Atyla	CEHFP	-	+
251004/36	Atyla	CEHFP	-	-
251004/37	Atyla	CEHFP	-	-
251004/38	Atyla	CEHFP	-	-
251004/39	Atyla	CEHFP	-	+
251004/40	Atyla	CEHFP	-	-
251004/42	Atyla	CEHFP	-	+
251004/50	Z-757	CEHFP	-	-
251004/51	Z-757	CEHFP	-	+
251004/52	Z-757	CEHFP	-	+
251004/53	Z-757	CEHFP	-	-
251004/57	Z-757	CEHFP	-	+
251004/58	Z-757	CEHFP	-	-
251004/71	303	CEHFP	-	+
251004/59	309	CEHFP	-	+
251004/60	309	CEHFP	-	+
251004/61	309	CEHFP	+	-
251004/62	309	CEHFP	-	+
251004/63	309	CEHFP	+	+
251004/64	309	CEHFP	-	-
251004/65	309	CEHFP	-	-
251004/70	309	CEHFP	-	+
251004/66	309	CEHFP	-	-
251004/72	309	CEHFP	-	+
251004/73	309	CEHFP	-	+
251004/74	309	CEHFP	+	+
251004/75	309	CEHFP	-	+
251004/76	Zinac	CEHFP	+	+
251004/77	Zinac	CEHFP	-	+
251004/78	Zinac	CEHFP	-	+
251004/79	Zinac	CEHFP	-	+
251004/80	Zinac	CEHFP	-	+
251004/81	Zinac	CEHFP	-	+
251004/82	Zinac	CEHFP	+	-

251004/83	Zinac	CEHFP	-	-
251004/84	Zinac	CEHFP	-	+
251004/85	Zinac	CEHFP	-	+
251004/86	Cherry	Santa Bárbara de Nexe	+	+
061204/3	Tyrade	CEHFP	-	-
061204/5	Tyrade	CEHFP	-	-
061204/6	Tyrade	CEHFP	-	-
061204/8	Tyrade	CEHFP	-	-
061204/10	Birloque	CEHFP	-	-
061204/11	Birloque	CEHFP	-	-
061204/13	Birloque	CEHFP	-	+
061204/14	Birloque	CEHFP	-	-
061204/15	Birloque	CEHFP	-	-
061204/17	Anastasia	CEHFP	-	-
061204/18	Anastasia	CEHFP	-	-
061204/24	Anastasia	CEHFP	-	-
061204/25	Encanto	CEHFP	-	-
061204/28	Encanto	CEHFP	-	-
061204/31	Encanto	CEHFP	-	-
061204/35	Bybal	CEHFP	-	-
061204/36	Bybal	CEHFP	-	-
061204/37	Bybal	CEHFP	-	-
061204/38	Bybal	CEHFP	-	-
061204/39	Bybal	CEHFP	-	-
061204/40	Bybal	CEHFP	-	+
061204/50	Atyla	CEHFP	-	-
061204/54	Atyla	CEHFP	+	+
061204/56	Atyla	CEHFP	-	+
061204/58	Atyla	CEHFP	-	-
061204/60	Z-757	CEHFP	-	+
061204/74	309	CEHFP	+	+
061204/75	Zinac	CEHFP	-	+
061204/76	Zinac	CEHFP	+	+
061204/77	Zinac	CEHFP	-	+
061204/78	Zinac	CEHFP	+	+
061204/79	Zinac	CEHFP	-	+
061204/80	Zinac	CEHFP	-	-
061204/81	Zinac	CEHFP	-	-
061204/82	Zinac	CEHFP	-	+
090205/24	Encanto	CEHFP	-	+
090205/25	Encanto	CEHFP	-	+
090205/26	Encanto	CEHFP	-	-
090205/27	Bybal	CEHFP	-	-
090205/28	Atyla	CEHFP	-	-
090205/29	Z-757	CEHFP	-	+
090205/30	309	CEHFP	+	-
090205/31	Zinac	CEHFP	-	+
090205/32	Zinac	CEHFP	-	-
090205/33	Zinac	CEHFP	-	+
090205/34	Zinac	CEHFP	+	-
090205/35	Zinac	CEHFP	-	+
090205/36	Zinac	CEHFP	-	-
090205/37	Zinac	CEHFP	-	+
241005/1	Tyrade	CEHFP	-	+
210705/32	D. Isabel	Campo Maior	-	+
210705/44	D. Joana	Campo Maior	-	-
210705/48	D. Joana	Campo Maior	-	+
210705/49	D. Joana	Campo Maior	-	+

210705/52	unknown	Campo Maior	-	+
210705/53	unknown	Campo Maior	-	+
241005/2	Tyrade	CEHFP	-	+
241005/3	Tyrade	CEHFP	-	-
241005/5	Birloque	CEHFP	-	+
241005/9	Birloque	CEHFP	-	-
241005/10	Anastasia	CEHFP	-	+
241005/11	Encanto	CEHFP	-	+
241005/13	Tybet	CEHFP	-	+
241005/14	Z-757	CEHFP	-	-
241005/16	309	CEHFP	-	-

Supplementary Table S2. Weed samples collected in this work in southern Portugal and dot-blot hybridization results. Digoxigenin-labeled probes specific to the IR of TYLCSV and TYLCV were used. *Sample codes are in the format DDMMYY/#. $CEHFP, Centro de Experimentação Horto-frutícola do Patacão, Faro.

Species	Sample code*	Location	Probe	
			TYLCSV	TYLCV
Solanum nigrum	311001/27	Estói	+	+
	070102/33	Estrada da Penha	-	-
	070102/36	Estrada da Penha	-	-
	110202/15	Estrada da Penha	-	-
	110202/16	Estrada da Penha	-	-
	210602/15	Braciais	-	-
	210602/16	Braciais	-	-
	210602/17	Braciais	-	-
	210602/18	Braciais	-	-
	300902/38	Caliços	+	+
	050303/5	Montenegro	-	-
	081004/37	Luz de Tavira	-	+
	210705/11	Campo Maior	-	+
	210705/17	Campo Maior	-	+
	210705/18	Campo Maior	-	+
	210705/19	Campo Maior	-	+
	210705/20	Campo Maior	-	+
	210705/10	Campo Maior	-	+
	210705/21	Campo Maior	-	-
Datura stramonium	150701/4	Ludo	-	+
Physalis ixocarpa	150701/5	Ludo	-	-
	310801/4	Ludo	-	-
	061105/2	CEHFP$	-	-
	061105/3	CEHFP	-	-
	061105/4	CEHFP	-	-
	061105/5	CEHFP	-	-
Salpichroa origanifolia	070102/45-B	Estrada da Penha	-	-
	070102/45-A	Estrada da Penha	-	-

Supplementary Figure S1. Phylogenetic network showing the relationships between parental (TYLCV and TYLCSV) and recombinant (TYLCMaV and TYLCAxV) genome sequences of the begomoviruses belonging to the TYLCV complex found in the Mediterranean basin. Neighbor-net analysis was carried out with SplitsTree 4[38]. The formation of a reticular network rather than a single bifurcating tree is suggestive of recombination (p = 0.0, PHI test).

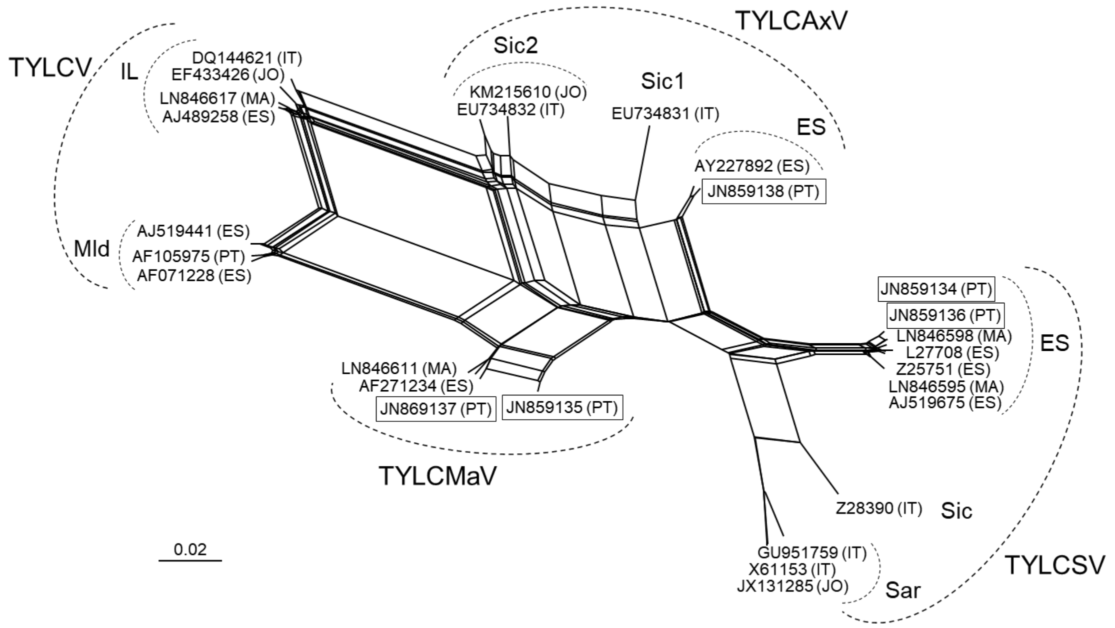

Supplement: Supplementary file 1 — Supplementary material [file 41598_2018_37971_MOESM1_ESM.pdf]
